# Randomized control trial of computer-based rehabilitation of spatial neglect syndrome: the RESPONSE trial protocol

**DOI:** 10.1186/1471-2377-14-25

**Published:** 2014-02-07

**Authors:** Thomas Van Vleet, Joseph DeGutis, Sawsan Dabit, Christopher Chiu

**Affiliations:** 1 Brain Plasticity Institute, 77 Geary Street, San Francisco, CA 94108, USA; 2 Boston Attention and Learning Laboratory, VA Boston Healthcare System, Department of Medicine, Harvard Medical School, 150 S. Huntington Ave., Boston, MA 02130, USA

**Keywords:** Hemispatial neglect, Rehabilitation, Non-spatial attention, Stroke, Computer-based cognitive training

## Abstract

**Background:**

Spatial neglect is a frequent and debilitating consequence of acquired brain injury and currently has no widely accepted standard of care. While previous interventions for spatial neglect have targeted patients’ overt spatial deficits (e.g., reduced contralesional visual scanning), far fewer have directly targeted patients’ non-spatial deficits (e.g., sustained attention deficits). Considering that non-spatial deficits have shown to be highly predictive of long-term disability, we developed a novel computer based training program that targets both sustained (tonic) and moment-to-moment (phasic) aspects of non-spatial attention (Tonic and Phasic Alertness Training, TAPAT). Preliminary studies demonstrate that TAPAT is safe and effective in improving both spatial and non-spatial attention deficits in the post-acute recovery phase in neglect patients. The purpose of the current trial (referred to as the REmediation of SPatial Neglect or *RESPONSE* trial) is to compare TAPAT to an active control training condition, include a larger sample of patients, and assess both cognitive and functional outcomes.

**Methods/Design:**

We will employ a multi-site, longitudinal, blinded randomized controlled trial (RCT) design with a target sample of 114 patients with spatial neglect. Patients will either perform, at their home, the experimental TAPAT training program or an active control computer games condition for thirty minutes/day, five days a week, over three months. Patients will be assessed on a battery of cognitive and functional outcomes on three occasions: a) immediately before training, b) within forty-eight hours post completion of total training, and c) after a three-month no-contact period post completion of total training, to assess the longevity of potential training effects.

**Discussion:**

The strengths of this protocol are that it tests an innovative, in-home administered treatment that targets a fundamental deficit in neglect, employs highly sensitive computer-based assessments of cognition as well as functional outcomes, and incorporates a large sample size (relative to other neglect treatment studies) in an RCT design.

**Trial registration:**

ClinicalTrials.gov identifier, NCT01965951

## Background

Approximately one half to two thirds of all patients with right hemisphere injury due to an acquired brain injury (ABI) exhibit a complex, debilitating array of neurological deficits known as the *spatial neglect syndrome* (referred to henceforth as neglect). Neglect refers to a collection of related spatial and non-spatial attention deficits that can occur after damage or disconnection to any number of interconnected cortical or subcortical areas (e.g., inferior parietal and frontal regions), usually in the right hemisphere [1-8]. While the severity of impairment can vary [9], the most obvious problem is that patients present with an inability to respond to stimuli on the contra-lesional or *neglected* side of space, often seemingly unaware that anything in that space exists [10]. Patients commonly suffer from poor navigation, bumping into objects or walls or disregarding potentially hazardous obstacles in the neglected field [5]. Patients with neglect also exhibit deficits in attention that are not spatially lateralized. For example, they often show deficits in alertness-based, time-challenged successive signal recognition, working memory, and sustained non-spatial attention [11-14].

Interestingly, non-spatial attention deficits are stronger predictors of chronic spatial neglect than are the visuo-spatial deficits themselves [12,13,15-17]. Furthermore, several recent studies and theoretical models indicate that poor regulation of intrinsic alertness can explain the severity of spatial bias in these patients [15,18-21]. In addition to these spatial and non-spatial attention deficits, reduced intrinsic alertness and impaired sustained attention can undermine more general cognitive functions such as memory and executive functions. By addressing patients’ non-spatial symptoms, it may be possible to not only improve their spatial biases, but also enhance higher-level cognitive functioning.

Several experimental therapies designed to remediate neglect have been developed over the last 30+ years. Behavioral treatments that target increased visual scanning in a top-down manner have shown some effectiveness, but may be overly time-consuming and may not be appropriate with patients who lack deficit awareness (i.e., anosognosia) [22]. Prism adaptation, a more bottom-up approach, has shown to improve leftward hypokinesis though it is unclear whether it can consistently improve more general neglect symptoms [23]. Mixed results have been found with pharmacological interventions that increase alertness, but two recent demonstrations with rotigotone and guanfacine suggest some benefit [24]. Finally, recent studies employing non-invasive brain stimulation (theta burst transcranial magnetic stimulation) to reduce activity in the intact hemisphere and improve functional outcomes have been quite promising [24].

Despite some evidence of benefits from these treatments, most largely fail to target patients’ non-spatial attention deficits. These non-spatial deficits are particularly important because they are significantly related to the chronicity of neglect. Pharmacological treatments may boost overall arousal/alertness levels but they have not shown to specifically improve moment-to-moment or sustained attention abilities in neglect patients. Thus, to address patients’ non-spatial deficits, we have developed a treatment targeting both sustained (tonic) and moment-to-moment (phasic) aspects of attention (tonic and phasic alertness training, TAPAT). TAPAT is a continuous performance task in which all stimuli are presented at central fixation (i.e., within a well-represented area of spatial awareness) with several key elements that help patients stay engaged such as jittered inter-trial intervals [25], rich, colorful, and novel stimuli, and a response inhibition component.

The results of our two previously published studies with approximately five hours of either visual or auditory TAPAT training over three weeks (10 sessions × 36 mins/session) are notable [26,27]. Not only did neglect patients demonstrate improvements in moment-to-moment and sustained attention after training, but they also showed more general improvements in sensitive measures of object-based attention and visual search. These initial studies of TAPAT demonstrate that enhancing non-spatial attention abilities can transfer to more general neglect symptoms (e.g., improvements in visuospatial search). The current multi-site clinical trial extends these studies with a longer duration of TAPAT training (8 weeks instead of 2 weeks), the inclusion of an active control training condition, a three-month follow-up visit to assess carryover effects, and the inclusion of additional computer-based, standard neuropsychological and functional outcome measures.

Two additional innovations in the protocol for the current clinical trial are notable. First, to our knowledge the current trial is the first, and perhaps the only Internet-based treatment for hemispatial neglect, designed to be completed in-home with minimal supervision. Of the eleven randomized clinical trials that have been completed in neglect (with adequate blinding) none have utilized computer-based cognitive training programs [28]. The advantages of computer-based training programs are that they can be performed at home via the Internet and, compared to pharmacological approaches, have minimal side effects (i.e., determined to be non-significant risk). Furthermore, computer-based training can be adaptively tailored to individual patient’s abilities to provide the appropriate amount of challenge. Finally, Internet-based training allows researchers and clinicians to remotely supervise training activities of multiple participants in real-time.

The second notable innovation in the current protocol relates to the use of several highly sensitive, reliable, and valid computer-based measures designed specifically for this population; that were not included in previous clinical trials. For example, the computer-based conjunction search task has shown to be a highly sensitive measure of goal-directed attention, revealing latent neglect symptoms in individuals who otherwise failed to show neglect on classic paper and pencil tasks [29]. These measures are not only more sensitive to residual impairments, but they also allow improved detection of training-related benefits, thereby enabling a more complete assessment of the extent to which patients’ deficits are partially versus fully addressed. These measures also facilitate better detection of neglect sub-types (i.e., behavioral profiling) to determine which individuals respond best to treatment. This is particularly relevant to neglect, as it is a very heterogeneous disorder.

### Aims and hypotheses

The aim of the current study is to test the effectiveness of 16 hours of computer-based TAPAT training to improve spatial neglect symptoms and enhance functional outcomes. Secondary aims include measuring the degree to which these effects persist after a three-month no-contact period and determining which patients best respond to TAPAT. Based on our previous findings, we predict that TAPAT will show large effect sizes, benefits will generalize across a wide range of neglect symptoms and functional outcomes, and these effects will persist beyond the three-month no-contact period. We hypothesize that those patients with the greatest impairments at baseline (i.e., most severe neglect), and those that demonstrate the most progress in treatment will show the greatest benefits.

## Methods/Design

### Overall design and timeline

**Figure 1:**
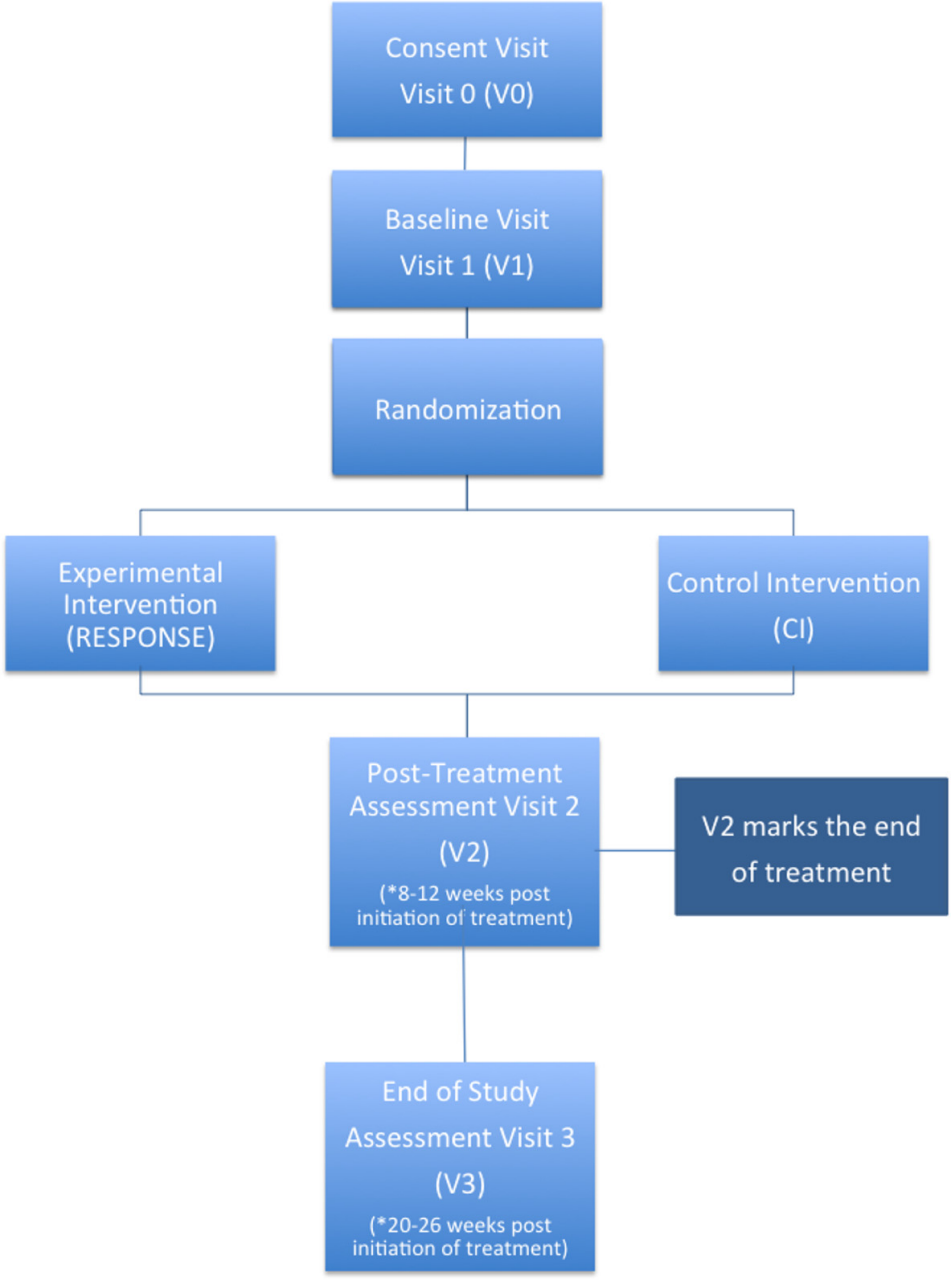
Study outline.

The current study will employ a multi-site, longitudinal, blinded randomized controlled trial (RCT) design with a target sample of 114 patients with neglect (see inclusion criteria below). Fifty-seven patients with neglect completing TAPAT will be compared to fifty-seven patients with neglect completing the active control condition (computer-based games; see Figure 1). Total participation time is approximately six months and includes four in-person assessment sessions. The first assessment session (V0) involves screening for eligibility (see inclusion/exclusion below). If the participant is eligible, they perform a baseline assessment (V1) to characterize their spatial and non-spatial attention, as well as cognitive and functional abilities before training. After the baseline assessment, patients are randomized to either the TAPAT or control training program and complete approximately twelve to sixteen weeks of in-home training, while monitored and coached by a research assistant (*cognitive remediation coach*). To measure potential training-related improvements, participants will be assessed within 48 hours after the completion of the total training (V2) by an assessor that is blind to group affiliation. To measure the persistence of potential training-related improvements, participants are also assessed after a three-month no-contact period (V3), again by an assessor blind to group affiliation. After this visit, participant activities are completed and trial participation ends.

### Study population

The study population is comprised of individuals with spatial neglect following an acquired brain injury (ABI), which may include stroke, tumor resection (without chemotherapy or radiation within 36-months) or other focal lesions.

Due to the average age of individuals with ABI (i.e., 60–90 years) and the unpredictable consequences of brain insult, we expect this population to have additional challenges, including but not limited to vision and/or hearing difficulties, motor difficulties (e.g., hemiakinesia, hemiparesis) and other unrelated, pre-morbid medical complications. We will only enroll individuals for whom these complications will not interfere with assessment procedures or completion of the training programs.

The following inclusion/exclusion criteria will be determined through our screening procedures during V0, which includes structured interviews, as well as computerized and standardized neuropsychological assessments of attention, cognition and functional abilities.

#### Inclusion criteria

1) Diagnosis of acquired brain injury will be confirmed via neuroimaging results from patients’ medical provider.

2) Diagnosis of Spatial Neglect will be confirmed by deficient performance on at least two of four common measures of Spatial Neglect:

● Mesulam cancellation task [30] (ages 50 and younger > 0 omissions; 51–80 > 4 omissions)

**Figure 2:**
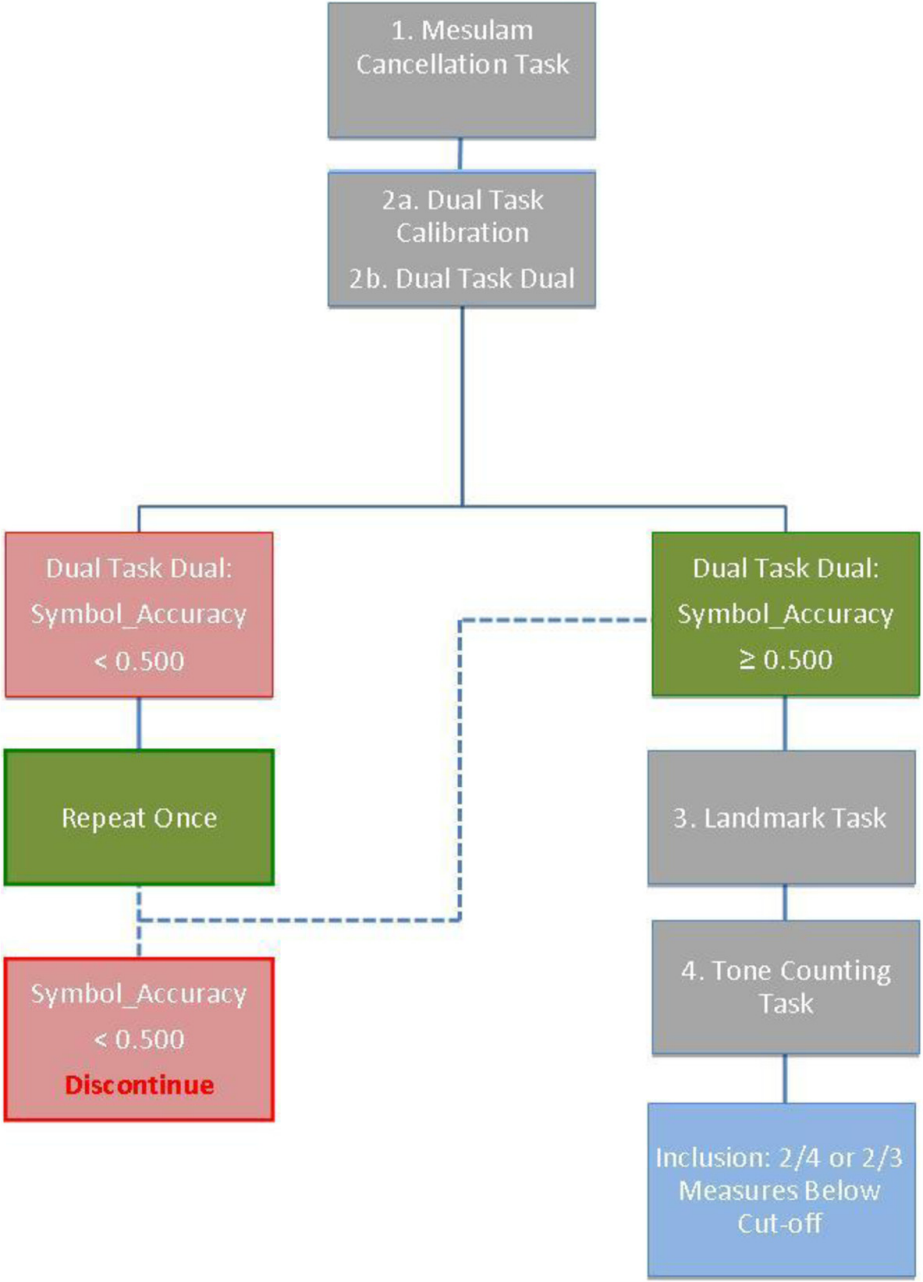
Spatial neglect screening protocol.

**Figure 3:**
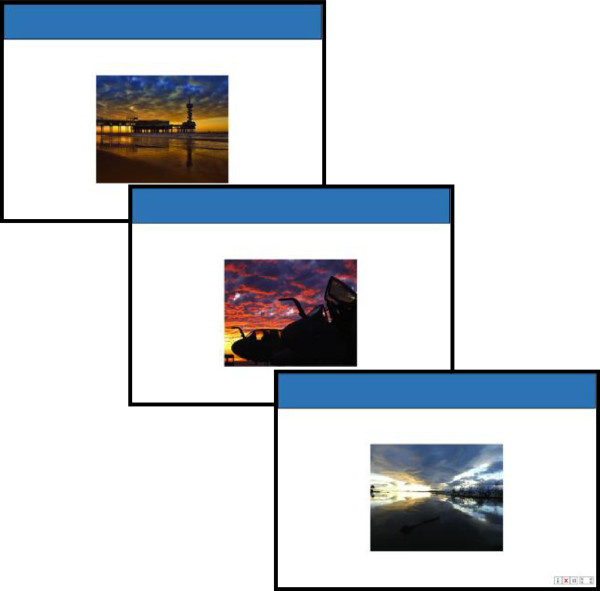
A visual, within-category version of TAPAT.

● Dual task (>19% difference in accuracy for right - left target trials) [31]. If performance on the Dual Task – Dual does not yield a reliable result, the task must be repeated. Upon repeating the task, if it still does not yield a reliable result, inclusion will be based on the performance on the remaining measures, where performance on two of the three must be consistent with neglect for the participant to be included in the study (Figures 2 and 3).

● Tone Counting task [13] (< 94% total accuracy)

● Landmark task (Rightward deviation from objective center significantly different from zero) [26].

3) 18 years of age or older at the time of consent.

4) At least three months post most-recent-brain-injury to minimize the effects of spontaneous recovery, as verified via participant self-report.

5) Fluent spoken English by the age of 12 in the judgment of the consenting clinician or as verified via participant interview.

6) Participants must have adequate sensorimotor capacity to participate in the trial, including visual capacity, auditory capacity, and motor capacity adequate to control a computer mouse.

#### Exclusion criteria

1) A conjunction of prior acquired brain injury *and* score > 8 on the Blessed Scale-Short form at Baseline (i.e., residual cognitive impairment or dementia) [32].

2) Diagnosis of severe depression according to the Beck Depression Inventory (BDI-II score >18) [33] at V0

3) Diagnosis of a chronic psychiatric disorder with associated cognitive impairments (e.g., schizophrenia) as confirmed through a structured clinical interview. Participants will not be excluded due to variation on this measure post-screening or worsening of depression.

4) Diagnosis of an illness, condition or treatment (within 36-months of consent) with known cognitive consequences (e.g., chemotherapy) verified through clinical interview.

5) Participants who have answered ‘yes’ to:

● Question 5 (Active Suicidal Ideation with Specific Plan and Intent) on the Columbia-Suicide Severity Rating Scale (C-SSRS) [34] or,

● ‘Yes’ to any of the suicide-related behaviors (actual attempt, interrupted attempt, aborted attempt, preparatory act or behavior) on the “Suicidal Behavior” portion will be excluded from the study if the ideation or behavior occurred within two months from Participant’s date of consent (as recommended by the FDA for treatment trials). Participants excluded for this reason will be referred for appropriate treatment. Further, participants meeting this criteria at any time throughout the study will be asked to complete a final assessment, if appropriate, then withdrawn from the study and referred for appropriate treatment.

6) Current or significant past history of substance abuse in the judgment of the Site PI.

7) Difficulty completing assessments and/or comprehending requirements of the trial (e.g., following verbal instructions).

8) Enrollment in a concurrent clinical trial involving an investigational pharmaceutical, nutraceutical, medical device or behavioral treatment that could affect the outcome of this study will be excluded. Participants will not be excluded for participation in conventional treatments (e.g., physical or occupational therapy) or use of prescribed medications.

9) Complete primary visual field deficit determined by scoring a ‘3’ (bilateral anopsia) on the NIH Stroke Severity Scale (NIHSSS) [35]:

a) 0 = no visual field deficit

a) 1 = partial hemianopsia

a) 2 = full hemianopsia

### Repeated assessment battery

Once a participant is deemed eligible for participation based on their V0 results, they are next then scheduled for their baseline session on the repeated assessment battery (V1), which takes approximately two hours. After completing this baseline assessment, participants are randomly assigned to either experimental or control training conditions (see below). Then, immediately after total training is completed, participants are given the repeated assessment battery (V2) and again after a three-month no contact period (V3).

The repeated assessment battery consists of a primary outcome measure (conjunction search task [29,36]), which measures the degree of bias in goal-directed spatial attention, and several secondary outcome measures. Secondary outcomes include sensitive assessments of spatial cognition and more global cognitive functioning as well as daily life measures of functional ability, quality of life, and sleep (Table 1).

**Table 1:** Primary and secondary outcome measures

**Domain**	**Measure**	**Primary/Secondary**
**Spatial attention**	Conjunction Search Task	Primary
**Spatial cognition**	Greyscales Task	Secondary
	Posner Cueing Task	Secondary
	Spatial Working Memory Task	Secondary
**Cognitive performance**	DKEFS-Verbal Fluency	Secondary
	WAIS-IV-Digit Span	Secondary
	Continuous Performance Task	Secondary
**Functional ability**	Catherine Bergego Scale	Secondary
	Barthel Index	Secondary
**Quality of life**	Short Form-12	Secondary
**Quality of sleep**	Pittsburgh Sleep Quality Index	Secondary

#### Spatial attention

**The primary outcome, spatial attention**, will be evaluated using the conjunction search task [26,27,29]. The conjunction search task (CS) requires participants to search for a target object amongst an array of distracters that share at least one feature (color or shape) with the target (e.g., a red square target located amongst red triangles and blue squares); see [26,27,29]. Thus, participants cannot simply search for the color or shape of the target object (i.e., the so-called ‘pop-out’ effect), but are required to engage in a serial search to find the unique *conjunction* of shape and color.

#### Spatial cognition

**The set of secondary outcome measures in the spatial cognition domain** is comprised of performance on the Greyscales task [37], Posner cueing task (see below), and spatial working memory (SWM) [38]*.* These measures have been empirically validated in this population and are thought to capture components of spatial cognition, including object-based attention (Greyscales [37]), reorienting (Posner), and the online representation of spatial locations.

Greyscales task captures perceptual bias by presenting two mirror-reversal objects progressively darkened on opposite ends of the object. Participants are then asked to report which gradient seems darker. Participants with Spatial Neglect may show a bias in judging the object darkened on the ipsilesional side as ‘darker’ than its mirror-reversed comparator on the majority of trials and the critical measure is the response index indicating Spatial Neglect.

Posner Cueing task [39] detects lateralized orienting deficits and requires participants to detect visual stimuli at attended or unattended peripheral locations while maintaining central fixation. Essentially, two stimuli are presented on left and right of the fixation point, and the subject is cued to attend to one. This task has been used widely in studies of Spatial Neglect, due to its sensitivity to detect mild cases.

Spatial Working Memory task detects impairment in spatial working memory capacity, which has been shown to correlate with severity of neglect [38]. Participants are presented with several possible target locations presented centrally along the vertical meridian. Following each trail, the participant is presented with a probe to a single location to which they must decide if the given location included in the possible target locations (yes/no).

#### Cognitive performance

**The set of secondary outcome measures in the cognitive performance domain** are comprised of tasks of working memory and executive function. We include these measures because executive functions have shown to correlate with functional ability in normal healthy populations [40]. According to principal components analysis by Miyake [41], executive functions can be broken down into the subdomains of working memory updating, switching and inhibitory control.

To capture working memory updating, we will use working memory span as an indication of updating efficiency (i.e., poor updating will result in shorter span, and vice versa). In particular, WAIS-IV Digit Span (forward, backward and sequencing) is a verbal working memory/span task, where the subject is given a list of numbers to remember and instructed to repeat the numbers either in the same order or in reverse order. This task was recently updated and now includes a Digit Span Sequencing (examinee is read a sequence of numbers and recalls numbers in ascending order) component to increase the working memory demands [42]. The total raw score for Digit Span is now based on all three components.

To capture switching ability, we will use the DKEFS verbal fluency subtest [43]. Scoring is based on accuracy per cue type (phonemic, semantic and category switching); response characteristics (e.g., repetitions) are also scored.

Inhibitory control will be assessed using a continuous performance measures with sound psychometric properties, good validity, generalizability across settings and adaptability for trials in patients with Spatial Neglect (similar to [44]). In general, this task requires sustained engagement (i.e., no inter-trial break), frequent responses and inhibitory control to over-come the proponent motor response.

#### Functional performance

**The set of secondary outcome measures in the functional performance domain** is comprised of performance on the Catherine Bergego Scale [45] and the Barthel Index [46]. We note that there is not a standardized, well-accepted functional assessment commonly used in Spatial Neglect treatment trials.

In this trial, we will employ one of each type: a directly observed performance measure designed to accurately capture performance in Spatial Neglect (Catherine Bergego Scale) [45], and a general clinical impression measure (the Barthel Index). We have chosen the Catherine Bergego Scale measure as the functional secondary measure due to its likely sensitivity in Spatial Neglect and its validity as a directly observed functional performance measure [45,47].

#### Quality of life

**A secondary outcome measure in the quality of life of domain** is comprised of performance on the Short-Form 12 (SF-12v2) [48], a measure of Health-Related Quality of Life. This assessment serves as a measure of the impact of program use on the participants’ own view of their impairment and function (i.e., quality of life).

#### Quality of sleep

**A secondary outcome measure in the quality of sleep domain** is comprised of performance on the Pittsburg Sleep Quality Index. This measure has been used in many outcome studies including a prior study of TAPAT outcomes in PTSD participants in which it effectively captured improvements in sleep quality post-training compared to a waitlist control group (DeGutis, in prep).

#### Residential status and daily activity

Exploratory analyses will be conducted on participants’ residential status and daily activity. Given the importance of independent living status in individuals with Spatial Neglect, we will also examine potential contribution of participants’ immediate post acquired brain injury and current residential status (e.g., assisted living facility, home assisted, home unassisted) and length of hospital stay immediately following insult (as well as re-admissions). We will also examine activities pre, post, and at follow-up.

### Randomization

Participants will be randomized after the baseline visit and before the planned first day of program use. All V0 and V1 data for each participant must be fully monitored, with all queries resolved, before randomization may take place. Given the potential influence of age and severity of spatial neglect on response to treatment, we will create four distinct strata for each site based on age (*Adults* 18–64 or *Seniors* ≥ 65 years of age) and severity of spatial neglect (conjunction search outcomes equal to or greater than 600ms difference (right threshold presentation time – left threshold presentation time) is considered moderate severe, and less than 600ms, moderate to mild neglect [29]): Adults-Severe neglect, Adults-Mild neglect, Seniors-Severe neglect, Seniors-Mild neglect. In particular, we will use a randomization server (http://www.sealedenvelope.com) that implements the specified procedure, and the Site Coordinating Center will issue a randomization assignment at the appropriate time, implementing an automated centralized group assignment procedure with allocation concealment.

### Blinding

Un-blinded Site Roles: At each site, cognitive remediation coaches are un-blinded in order to provide support for participants using their assigned programs. They will be distinct from staff administering and scoring assessments. Additionally, site sub-investigators authorized to register participants within the RESPONSE system will remain un-blinded and may not participate in the assessment, evaluation, or follow-up of study participants.

Blinded Site Roles: All site staff responsible for the administration and scoring of participant assessments will remain blinded to participant treatment. Site Principal Investigators will be required to complete a Delegation of Authority Form prior to the start of the study, indicating which activities individual site research team members will be authorized to complete. Site Principal Investigators will also remain blinded.

To prevent un-blinding, the following controls will occur at the site level:

1. The treatment condition and the control condition will be identified as “Treatment A” and “Treatment B”;

2. Participants will be reminded not to discuss details related to treatment with psychometricians and/or clinical evaluators during the informed consent process as well as prior to initiation, and at the conclusion of, each assessment visit;

3. Site personnel will be instructed to **not** discuss details of either treatment arm during open participant groups or forums;

4. Sites will be required to execute the protocol in a manner that minimizes the possibility of accidental un-blinding of psychometricians or clinical evaluators (e.g., unintended viewing of treatment sessions);

5. Sites will be asked to post signage in appropriate areas throughout the facility reminding staff and participants to **not** discuss treatment details in open locations.

At the halfway point of the trial and at the end of the trial, psychometricians will be asked questions designed to evaluate the integrity of the blinding procedures employed throughout the study

### Description of treatment program

*The Experimental Treatment Program incorporating TAPAT* is a computerized cognitive remediation program consisting of a set of specific cognitive exercises. Participants perform hundreds of trials over the course of a session, with auditory and visual feedback and rewards to indicate if the trial was performed correctly or incorrectly. After each session, the difficulty of the next session is updated (e.g., less inter-stimulus-interval jitter) to ensure that each participant is appropriately challenged. Summary screens including game metrics (points, levels) and exercise metrics (usage, progress) are shown to the participant at the end of each session.

There are multiple cognitive exercises in the experimental treatment program comprised of the core exercise, TAPAT. A description of exercises in experimental treatment program is as follows:

•*Tonic and Phasic Alertness Treatment (three variations):* The goals of this exercise are to improve the individual’s intrinsic regulation of alertness and their executive control: ability to sustain attention and respond to successively presented stimuli in a consistent manner (i.e., low RT variability), and ability to inhibit the proponent motor response when a target is presented. Exercise variants: 1) a visual category-nonspecific version; 2) a visual, within-category version; 3) a visually engaging, category-nonspecific version.

All training sessions will consist of the three variations of TAPAT to provide variety during each training session. A summary of the training schedule is provided below.

### Training schedule

Session 1: Embedded Assessments (32 min/session)

Session 2–19: TAPAT > TAPAT > TAPAT (32 min/session)

Session 20: Embedded Assessments (32 min/session)

Session 21–38: TAPAT > TAPAT > TAPAT (32 min/session)

Session 39: Embedded Assessments (32 min/session)

*Active Control Program (commercially-available computer games):* The active control program is composed of 12 commercially available computer games and is designed to: 1) be a face-valid approach to cognitive remediation in Spatial Neglect (analogous to crossword puzzles for age-related cognitive decline), ensure that participants remain blind to group affiliation, and match the experimental treatment program in halo or expectation-based influence on performance in neuropsychological outcome measures; 2) match the experimental treatment program in overall program use intensity, time-spent attending, delivered rewards, and overall engagement; and 3) provide a comparison group that matches the experimental treatment group on the aforementioned attributes, but without the known therapeutic elements.

### Data analysis

For the main analysis, we will define an Intent-To-Treat (ITT) population that includes all participants who complete a computer set-up visit. We will compare treatment and active control groups in the ITT population to determine if any differences in baseline demographic, characterization, outcomes variables, or time of total program use remain after the randomization process. We will test the following hypotheses, in addition to exploratory analyses: 1) Primary outcome – experimental treatment versus active control improves spatial attention, 2) Secondary outcome – experimental treatment versus active control improves spatial cognition, 3) Secondary outcome – experimental treatment versus active control improves cognitive performance, 4) Secondary outcome – experimental treatment versus active control improves functional performance and 5) Secondary outcome – experimental treatment versus active control improves quality of life. 6) Secondary outcome – experimental treatment versus active control improves quality of sleep. To examine each hypothesis, we will examine the data from each outcome measure(s) associated with the Primary or Secondary outcomes using a linear mixed-effects model with group and time as fixed factors, site as a random factor, and additional factors/covariates as required if there are trends towards significant baseline differences (p < 0.1) in the treatment and active control groups. Missing data will be handled with an iterative maximum likelihood procedure to optimally estimate model parameters. The key value for significance will be the group-by-time interaction term. This modeling will be conducted with a Type I error set at 0.025 for each model.

To test the hypothesis that the experimental treatment program versus the active control program results in an enduring effect, we will use the same modeling approach based on the pre-training assessment (baseline) and end of study (follow-up) assessment data using a mixed-effects model parallel to those used to meet the primary and secondary aims. Finding a significance level of p < 0.025 on all co-primary measures will support the hypothesis that the training drives persistent attention as well as executive and real-world functional improvements in this participant population above and beyond an active control.

Secondary, exploratory analysis will test for effects in exercise-based progress as well as by domain (e.g., working memory, attention, processing speed) to determine the unique benefit in each area of attention, cognition and independent function. First, to determine if the treatment effect varies based on participants’ clinical severity of neglect (Conjunction search difference score: Left TPT – Right TPT) or symptom subtype (e.g., sensory loss vs. intact sensory systems), we will correlate neglect severity (or subtype(s)) with gain scores on composite attention or executive performance and composite functional performance (as well as individual assessment measures). Additionally, we will measure whether improvements on certain components of the training program (e.g., improvements at inhibiting one’s response to target stimuli) are associated with composite attention, executive or functional performance scores (as well as individual assessment measures). This could indicate whether certain patterns of training improvements are related more to improvements in attention, executive or functional performance. These analyses will be evaluated after correcting for multiple comparisons (i.e., Bonferroni).

## Discussion

Despite experimentation with dozens of treatments for spatial neglect over the last 50 years [49], there still does not exist an effective, widely accepted treatment approach for this severely debilitating disorder. The current RESPONSE protocol will help determine whether TAPAT is effective in creating lasting deficit reduction and functional improvements and will potentially provide the building blocks for the further development and dissemination of this treatment, ultimately leading to an improved standard of care for neglect.

### Strengths

One strength of TAPAT is that it targets fundamental deficits in non-spatial attention common to nearly all chronic neglect patients. Attesting to this, our previous results have shown that TAPAT is effective in improving spatial and non-spatial attention for a wide range of neglect patients (especially the most severely impaired). In addition, TAPAT is simple to perform, easy to remotely monitor (e.g., by a clinician), and is well tolerated by patients. Because of these qualities, TAPAT can be readily administered to many patients (with potential for remote web-based administration) with fewer burdens on the clinician than other neglect therapies (e.g., scanning training or prism adaptation).

According to a recent systematic review of cognitive rehabilitation for spatial neglect issues with study bias, sample size and longitudinal effects rendered many approaches ineffective [28]. The current study is blinded and deals with appropriate allocation concealment. Moreover, this protocol will measure immediate as well as lasting benefits of treatment. Lastly, many previous studies lacked statistical power due to a small sample size. RESPONSE will have one of the largest sample sizes of neglect treatments studies, with an aim of 114 participants.

### Weaknesses

There are also potential limitations. Due to the long duration and multiple components of our study, attrition rates may pose a potential limitation. Furthermore, one of the common symptoms of neglect is a lack of motivation, which could prevent timely completions of the self-initiated training program. With a standardized protocol, frequent check-ins, and regular feedback from our research assistants, we aim to have a limited dropout rate.

### Trial status

The trial is currently in the recruitment phase.

### Ethics and human research

The Western International Review Board (WIRB) is designated to review and provide continuing oversight of ethical standards involving human subjects research (WIRB Pro Number 20132014). Research is conducted in accordance with the Declaration and Helsinki and monitored by the WIRB. Participants interested in the study will meet with qualified study staff for the consenting process, during which the participant is informed of the nature of the trial, purpose of research, trial procedures, risks and benefits, confidentiality, etc. Following consent, the participant will be assessed for eligibility and potential enrollment in the trial. Minors are excluded from this study and will not undergo the consenting process.

## Abbreviations

ABI: Acquired brain injury; BDI: Beck depression inventory; C-SSRS: Columbia- suicide severity rating scale; CS: Conjunction search task; ITT: Intent-to-treat; NIHSS: NIH stroke severity scale; RESPONSE: Rehabilitation of spatial neglect syndrome; SF-12v2: Short form 12; SWM: Spatial working memory; TAPAT: Tonic and phasic alertness treatment.

## Competing interests

TV and JD have a patent application based around the TAPAT procedure (PCT/US11/042031). CC and SD declare that they have no competing interests.

## Authors’ contributions

TV and JD conceived the study and drafted the manuscript. CC and SD helped with manuscript preparation and SD assisted in past and current neglect research studies, study coordination and data management. All authors read and approved the final manuscript.

## Pre-publication history

The pre-publication history for this paper can be accessed here:

http://www.biomedcentral.com/1471-2377/14/25/prepub
